# Dietary Protein Sources and Muscle Mass over the Life Course: The Lifelines Cohort Study

**DOI:** 10.3390/nu10101471

**Published:** 2018-10-10

**Authors:** Nikita V. Alexandrov, Coby Eelderink, Cécile M. Singh-Povel, Gerjan J. Navis, Stephan J. L. Bakker, Eva Corpeleijn

**Affiliations:** 1 Department of Epidemiology, University Medical Center Groningen, University of Groningen, 9713GZ Groningen, The Netherlands; e.corpeleijn@umcg.nl; 2 Division of Nephrology, Department of Internal Medicine, University Medical Center Groningen, University of Groningen, 9713GZ Groningen, The Netherlands; c.eelderink@umcg.nl (C.E.); g.j.navis@umcg.nl (G.J.N.); s.j.l.bakker@umcg.nl (S.J.L.B.); 3 FrieslandCampina, 3800BN Amersfoort, The Netherlands; Cecile.Singh-Povel@frieslandcampina.com

**Keywords:** protein intake, animal protein, plant protein, dairy protein, sarcopenia, muscle mass, creatinine excretion, nutrition, physical activity, adults

## Abstract

The influence of dietary protein intake on muscle mass in adults remains unclear. Our objective was to investigate the association between protein intake and muscle mass in 31,278 men and 45,355 women from the Lifelines Cohort. Protein intake was estimated by food frequency questionnaire and muscle mass was estimated from 24 h urinary creatinine excretion. The age range was 18–91 years and mean total protein intake was 1.0 ± 0.3 g/kg/day. Across increasing quartiles of total protein intake, animal protein intake, and fish/meat/egg protein intake, creatinine excretion significantly increased in both men (+4% for total and +6% for fish/meat/egg protein intake, *p* < 0.001) and women (+3% for total and +6% for fish/meat/egg protein intake, *p* < 0.001). The associations were not systematically stronger or weaker with increasing age, but associations were strongest for young men (26–45 years) and older women (>75 years). The association between total protein intake and muscle mass was dependent on physical activity in women (*p* interaction < 0.001). This study suggests that total protein intake, animal protein intake, and in particular fish/meat/egg protein intake may be important for building and preserving muscle mass. Dietary protein sources should be further studied for their potential to build and preserve muscle mass.

## 1. Introduction

Sarcopenia is the age-related loss of muscle mass and strength [1]. Muscle mass is lost at a rate of 3–8% per decade after the age of 30 [2]. Sarcopenia is associated with greater risk of falls, disability, loss of independence, and mortality [1,3]. Loss of muscle mass and strength can increase the risk for developing chronic conditions, such as osteoporosis [4]. Therefore, gaining and/or preserving muscle mass is vital not just to older adults, but also to younger adults.

There is significant variability between individuals in rates of muscle loss, some of which can be explained by fixed factors (such as sex, body size, heritability, and early life environment) while the remaining variability remains unexplained [5]. Therefore, there is increasing interest in the influence of lifestyle, especially of modifiable factors such as diet [6] and physical exercise [7] on sarcopenia in older adults.

Dietary protein intake, in particular, has been implicated as an important contributor to the maintenance of muscle mass and strength, and previous observational studies have shown that inadequate dietary protein intake may accelerate loss of muscle, leading to sarcopenia [8,9,10,11,12,13,14]. Many older adults may not be consuming adequate amounts of dietary protein, particularly institutionalized elderly [15]. The Recommended Dietary Allowance (RDA) for dietary protein intake in the US and Canada is 0.8 g/kg/day [16]. The Netherlands Nutrition Center provides the same RDA for protein intake. It should, however, be noted that this RDA does not increase with age, while experts in the field of protein and aging recommend protein intakes between 1.2 and 2.0 g/kg/day for elderly adults to maintain optimal muscle health [17,18].

Besides quantity, the quality of protein is of importance. Previous observational studies suggest that the quality of protein, determined by its essential amino acid composition, digestibility, and bioavailability of amino acids [19], is an important determinant of its potential to affect muscle mass and strength [8,9,10]. According to data from the Health, Aging, and Body Composition study, animal protein intake, but not plant protein intake, was associated with changes in lean mass in older adults [8]. Sahni et al. [10] examined associations between protein intake, lean mass of the legs, and quadriceps strength in a community-based cohort of adult men and women (29–86 years). In this cohort, total protein and animal protein intake, but not plant protein intake, were positively associated with lean mass of the legs in men and women. These studies did not address, however, whether the source of animal protein, i.e., dairy, meat or eggs makes a difference. Meat is a particularly good source of high quality proteins, which not only provides a large quantity of essential amino acids, but also other biologically active compounds such as creatine, carnitine, conjugated linoleic acid, iron, cobalamin, and vitamin B12 [20]. Specific components in dairy products, such as the high amount of branched-chain amino acids in whey protein, are also suggested to have positive effects on muscle mass [21]. In an observational study, Radavelli-Bagatini et al. [11] found a positive association of dairy consumption with muscle mass and function in elderly women aged 70 to 85 years, suggesting that intake of dairy products could be a modifiable lifestyle factor in reducing sarcopenia with aging.

Physical inactivity or decreased physical activity is another factor driving sarcopenia and therefore physical activity can be regarded as an important factor to build and preserve muscle mass [7]. The effectiveness of exercise training in building and preserving muscle mass has been documented by many studies [22,23,24]. Furthermore, intervention studies have clearly demonstrated that combining physical exercise with protein intake seems to have a positive synergistic effect on muscle protein synthesis [15,25]. Therefore, intervention studies suggest that dietary protein should be prescribed together with physical exercise in order to optimize muscle health [26].

Even though several intervention studies on the role of protein exist, these studies have only been conducted in specific target groups, primarily elderly [15,25]. Population-based studies, i.e., observational studies in a larger group on the role of dietary protein on muscle are sparse. Furthermore, the conducted observational studies did not stratify by age groups, and tended to have small sample sizes [8,9,10,12]. Consequently, it is difficult to draw inferences to the general population. The aim of the present study was, therefore, to examine the strength of the associations between intakes of different dietary protein sources and estimates of muscle mass and how this association depends on age and physical activity in 76,633 men and women aged 18–91 years from the Lifelines Cohort Study.

## 2. Materials and Methods

### 2.1. Study Population

Data for this analysis were from the Lifelines Cohort Study. Lifelines is a multi-disciplinary prospective population-based cohort study examining the health and health-related behaviors of 167,729 persons living in the north of The Netherlands. It employs a broad range of investigative procedures in assessing the biomedical, socio-demographic, behavioral, physical and psychological factors which contribute to the health and disease of the general population, with a special focus on multi-morbidity and complex genetics. The Lifelines adult study population is broadly representative for the adult population of the north of The Netherlands [27]. Detailed information on the cohort profile can be found in previous publications [28,29].

To perform this study, a subset of the cross-sectional data from the Lifelines Cohort Study was used. This data was collected between 2006 and 2016. The subjects analyzed were participants with completed Food Frequency Questionnaires (FFQ) (*n* = 77,992). Subjects in which no urinary creatinine excretion could be calculated (*n* = 320) were excluded. Subjects in which data on either sex, age, height, or weight were missing, were also excluded (*n* = 24). Furthermore, like in previous studies [8,10,11,30,31] participants who reported extremely low or high values for energy intake (<500 kcal/day or >3500 kcal/day for women and <800 kcal/day or >4000 kcal/day for men) (*n* = 1015) were also excluded. The final sample comprised of 76,633 participants in the age range of 18–91 years.

All participants provided written informed consent before participating in the study. The Lifelines Cohort Study is conducted according to the principles of the Declaration of Helsinki and in accordance with the research code of the University Medical Center Groningen (UMCG). The Lifelines study is approved by the medical ethical committee of the UMCG, The Netherlands (ethic approval code: METc 2007/15).

### 2.2. Estimation of Muscle Mass

A widely accepted method for estimation of total-body skeletal muscle mass is assessment of excretion of creatinine in a collection of 24 h urine [32]. In healthy adults, creatinine is produced by non-enzymatic dehydration of intracellular creatine and phosphocreatine from muscle tissue at a constant rate (around 1–2% per day) [32]. This metabolic end product is then released into extracellular fluid and excreted unchanged in urine. Twenty four hour urinary excretion of creatinine is considered a reliable measure of muscle mass even in patients with advanced renal failure, in children and adolescents, and in elderly people [32,33,34,35]. However, urinary creatinine excretion is influenced to some extent by dietary intake of creatine (derived from meat) and by protein intake [32,36]. Creatinine excretion was calculated by multiplying creatinine concentration in the urine with volume of the urine of a single 24 h urine collection. Creatinine in urine was determined using the Jaffé method on a Roche Modular P chemistry analyzer (Roche, Basel, Switzerland).

### 2.3. Dietary Assessment

A self-administered FFQ was used to estimate habitual nutrient intake of 110 food items during the last 4 weeks. The FFQ was derived from an existing validated Dutch FFQ [37,38] and optimized to estimate energy intake and macronutrients, including protein, fat, carbohydrate, and alcohol intake. For 46 main food items, frequency of consumption was indicated as ‘not this month’ or in days per week or month; including the amount (in units or specified portion size) consumed each time. The FFQ also included 37 questions on consumption of sub-items (e.g., types of cheese like 20+/30+ cheese, 40+ cheese, 48+ cheese, or cream cheese) for which frequency was specified as never, sometimes, often and (almost) always. The FFQ was analyzed for macronutrients using the 2011 Dutch food composition table (NEVO). Protein intake was defined as total protein, animal protein (divided into dairy protein and fish/meat/egg protein), and plant protein, all in grams per day. Protein intake was examined using the nutrient residual energy-adjusted model, in which the protein intakes of the participants are regressed on their total energy intakes [39].

### 2.4. Demographics, Lifestyle Factors, and Blood Biomarkers

Information on age, sex, education, smoking status, alcohol consumption, physical activity, and chronic disease history was obtained via the self-administered questionnaires at baseline. A fixed staff of well-trained technicians, who had a long experience in the clinical practice, used a standardized protocol to obtain blood pressure and anthropometric measurements: height, weight, and waist circumference. Anthropometric measurements were taken in light clothing and without shoes. Body weight was measured to the nearest 0.1 kg. Height and waist circumference were measured to the nearest 0.5 cm. Height was measured with a stadiometer placing their heels against the rod and the head in Frankfort Plane position. Waist circumference was measured in standing position with a tape measure all around the body, at the level midway between the lower rib margin and the iliac crest. Body surface area (BSA) was calculated using Dubois’ formula: BSA (m^2^) = 0.007184 × height (cm) 0.725 × weight (kg) 0.425 [40]. Body mass index (BMI) was calculated as weight (kg) divided by height squared (m^2^). Education level was categorized as low (no formal education, only primary school or intermediate vocational education), medium (higher secondary education) or high (higher vocational education and university). Smoking status was categorized as non-smoker, former smoker, and current smoker. Alcohol consumption was categorized as 0 g/day, >0–<10 g/day, ≥10–<20 g/day, ≥20 g/day. Physical activity was assessed by the validated SQUASH questionnaire (“Short QUestionnaire to ASsess Health-enhancing physical activity”) [41]. The SQUASH estimates physical activities pre-structured in sports, commuting-, leisure time-, and household activities, and activities at work or school, referring to a normal week in the preceding months. Questions included type of activity, frequency, duration and intensity. Metabolic equivalent (MET) values were assigned to activities as defined by the Ainsworth’ compendium of Physical activities [41]. One MET unit is defined as the energy expenditure for sitting quietly. Activities with a MET value of 2 to <4 were classified as light, 4 to <6.5 as moderate, and ≥6.5 as vigorous intensity. A moderate-intensity and vigorous-intensity physical activity (MVPA) score was calculated by multiplying duration (minutes per week) with the MET value. 

Blood was collected in the fasting state, between 08:00 and 10:00 in the morning. On the same day, serum levels of cholesterol were measured using an enzymatic colorimetric method on a Roche Modular P chemistry analyzer (Roche, Basel, Switzerland). Fasting blood glucose was measured using a hexokinase method.

Missing values on prevalent health behaviors (i.e., physical activity (7.3%) and smoking status (0.4%)), prevalent health conditions (i.e., diabetes (0.2%), ischemic heart disease (1.2%), congestive heart failure (1.4%), cerebrovascular accident (0.5%), chronic obstructive pulmonary disease (COPD) (0.4%), cancer (0.1%), and hypertension (0.1%)), hemoglobin (0.5%), cholesterol (0.30%), glucose (0.57%), and lymphocyte percentage (2.2%) were imputed using single imputation.

### 2.5. Statistical Analyses

Descriptive statistics are reported as mean ± standard deviation (SD), *n* or percentages. Protein intake was modeled as a continuous variable and categorical variable by using sex-stratified quartiles. By using the nutrient residual energy-adjusted model, the sex-stratified quartiles of residual protein represent the variability in absolute protein intake for participants with the same total energy intake.

Quartile group differences in the means of continuous variables were tested by univariate analysis of variance (ANOVA). The chi-square test was used to evaluate any differences between quartiles in the qualitative variables. Stepwise multivariable linear regression was used to examine the associations between protein intake and creatinine excretion by calculating regression coefficients (β), estimating the difference in creatinine excretion as a marker of muscle mass associated with 1 unit increase in protein intake. Seven age groups were created to examine the association between protein intake and creatinine excretion over the life course: 18–25, 26–35, 36–45, 46–55, 56–65, 66–75, >75. An analysis of covariance (ANCOVA) was used to conduct all pairwise comparisons of least square adjusted creatinine excretion by quartiles of protein intake and to test for a linear trend across quartiles. All regression models were initially adjusted for age and BSA. Animal protein and plant protein were included in the same regression model to adjust for each other. Additional models were also adjusted for total energy intake, prevalent health behaviors (i.e., smoking status, alcohol consumption, and physical activity), health conditions (i.e., diabetes, ischemic heart disease, congestive heart failure, cerebrovascular accident, COPD, and cancer), hemoglobin, and lymphocyte percentage. Posthoc comparisons were made by Bonferroni test for ANCOVA.

Possible interactions between protein intake and age, sex and physical activity were tested through the inclusion of a cross-product term in linear models and visualized through stratified analyses. 

All analyses were conducted using IBM SPSS Statistics version 22 (IBM Corporation, Armonk, NY, USA). All *p* values are 2-tailed. A nominal 2-sided *p* value of 0.05 was considered statistically significant for all analyses. *p*-trend was considered significant when <0.05 and considered marginally significant when <0.10.

## 3. Results

### 3.1. Subject Characteristics

The baseline characteristics of the participants by sex-stratified quartiles of energy-adjusted total protein intake are shown in Table 1. The mean age of the participants was 44.9 ± 12.8 (range: 18–91 years), 59.2% were women, mean total protein intake (1.0 g/kg/day) was higher than the Dutch recommended amounts of 0.8 g/kg/day. Participants with a higher total protein intake were older, had lower intakes of alcohol, were less likely to be current smokers and more likely to be former smokers. In addition, those with a higher total protein intake were more likely to be physically active, more likely to have diabetes, congestive heart failure, hypertension, cancer, a higher weight, BMI, and waist circumference, and a higher urinary creatinine excretion (used as a marker for muscle mass) (*p* < 0.001). 

### 3.2. Associations between Protein Intake and Creatinine Excretion

Associations between protein intake and creatinine excretion were examined using both categorical (Table 2) and continuous protein intake (Table 3), for men and women separately.

Adjusted least square means for creatinine excretion among the different quartiles of energy-adjusted protein intake are shown in Table 2. Across quartiles of total protein (*p* for trend: <0.001), total animal protein (*p* for trend: <0.001), and fish/meat/egg protein intake (*p* for trend: <0.001) creatinine excretion increased in both men and women. Across quartile increments of plant protein and dairy protein intake, creatinine excretion did not increase. When model 1 was subsequently adjusted for potential confounders (model 2), the observed associations remained significant (*p* for trend: <0.001). Similar associations were observed when protein intake was examined as a continuous variable (Table 3). Plant protein was marginally associated with creatinine excretion in women (*p* = 0.091) (model 1), but when model 1 was subsequently adjusted for potential confounders (model 2), this association attenuated and was not associated with creatinine excretion. 

### 3.3. Age Groups

Adjusted regression coefficients per unit of sex-stratified energy-adjusted protein intake for creatinine excretion among the different age groups are shown in Table 4. 

In men, total protein, total animal protein, and fish/meat/egg protein intake were positively associated with creatinine excretion, except in the >75 age group. Dairy protein intake was positively marginally associated with creatinine excretion in the 56–65 age group and plant protein was positively associated with creatinine excretion in the 26–35 age group. The positive marginal association for dairy protein intake disappeared after adjusting for potential confounders (model 2).

In women, total protein, total animal protein, and fish/meat/egg protein intake were positively associated with creatinine excretion in all age groups. Dairy protein was positively associated with creatinine excretion in elderly women (66–75 years). Plant protein was positively marginally associated with creatinine excretion in middle aged women (46–55 years), elderly women (66–75 years) and positively associated in women aged >75. After adjusting for potential confounders, similar associations were observed for total protein, total animal protein, and fish/meat/egg protein intake, but the positive associations for dairy protein and plant protein intake attenuated and were no longer significant.

### 3.4. Physical Activity

There was a significant interaction between total protein intake and physical activity in women (*p* = 0.001), but not in men (*p* = 0.473). Similarly, there was a significant interaction between total animal protein intake and physical activity in women (*p* = 0.012) but not in men (*p* = 0.267). By contrast, there was no significant interaction between plant protein intake and dairy protein intake and physical activity in either men or women. Finally, there seemed to be a trend for interaction between fish/meat/egg protein intake and physical activity in women (*p* = 0.072), but not in men (*p* = 0.104).

The strength of the association between total protein intake and creatinine excretion did not increase linearly across quartile increments of physical activity, however. When women were stratified according to their MVPA score into quartiles, the following associations between total protein intake and creatinine excretion were observed: Q1. β: 0.017 (0.013–0.021), *p* < 0.001; Q2. β: 0.019 (0.015–0.024), *p* < 0.001, Q3. β: 0.015 (0.011–0.020), *p* < 0.001, Q4. β: 0.008 (0.004–0.012), *p* < 0.001 (with Q1 representing the lowest MVPA score and Q4 representing the highest MVPA score). Results were similar for total animal protein and fish/meat/egg protein intake (data not shown). Results were similar in fully adjusted models (data not shown). 

## 4. Discussion

This study examined cross-sectional associations between protein intake and estimated muscle mass in a large, wide-age range cohort of adult men and women. Additionally, it was investigated how age and physical activity would influence these associations. Overall, total protein, total animal protein, and fish/meat/egg protein intake, but not plant protein and dairy protein intake, were positively associated with creatinine excretion, also after adjusting for potential confounders. Plant protein intake was positively (marginally) associated with creatinine excretion in younger men (26–35 years), middle-aged (46–55 years) and older (>66 years) women and dairy protein was positively associated with creatinine excretion in older (66–75 years) women, although all of these associations attenuated and were no longer significant after adjusting for potential confounders. Physical activity influenced the association between total protein intake and creatinine excretion in women, but not in men.

Previous observational studies of dietary protein intake and muscle mass have shown mixed results. Houston et al. [8] reported that older community-dwelling adults (70–79 years) in the highest quintile of energy-adjusted protein intake lost ~40% less lean muscle mass and appendicular lean mass than those in the lowest quintile of protein intake over a period of 3 years. In their study, animal protein intake, but not plant protein intake, was associated with changes in lean mass. Sahni et al. [10] examined associations between protein intake, lean mass of the legs, and quadriceps strength in a community-based cohort of adult men and women (29–86 years). Additionally, in this cohort, total protein and animal protein intake, but not plant protein intake, were positively associated with lean mass of the legs in men and women. The results from the present study are consistent with these previous studies. By contrast, Huang et al. [9] examined the association between protein intake and low muscle mass among the community-dwelling elderly (65–85 years) population in Northern Taiwan and found that estimated skeletal muscle mass increased significantly across quartiles of total protein density and vegetable protein density. In their study, however, only total protein and vegetable protein intake were investigated and they were not compared to animal protein intake. Radavelli-Bagatini et al. [11] examined the association of dairy intake with body composition in older community-dwelling women (70–85 years) and found an association of higher dairy intake with greater whole body lean mass. In the present study, a positive association between dairy protein intake and creatinine excretion was found in women aged 66–75 only, which attenuated and was no longer significant after adjustment for potential confounders. The >75 age group in the present study, as compared to the other groups, was likely too small to show a significant difference. The study of Radavelli-Bagatini et al. [11], examined total dairy intake, whereas in the present study, total dairy protein intake was examined.

In 2013, the Food and Agriculture Organization (FAO) released a report [42] proposing a new method, which better reflects current scientific understanding, for assessing dietary protein quality. This new method, the Digestible Indispensable Amino Acid Score (DIAAS), addresses limitations of the old Protein Digestibility Corrected Amino Acid Score. The DIAAS emphasizes, among other, individual amino acid concentration and digestibility at the end of the small intestine, as opposed to crude protein digestibility over the entire digestive tract. This method should enable forming a better distinction between high and low quality protein sources. Sufficient research data is required to change current practice, however [43]. While the present study did not evaluate protein sources based on an individual amino acid basis, the epidemiological approach employed suggests that not only the quantity, but also the source of the ingested protein is important in promoting muscle mass. As described previously, the quality of a protein is determined by its essential amino acid profile, digestibility, and bioavailability of amino acids [19,44]. Protein from animal sources, as compared to protein from plant sources, provides all the essential amino acids, has a higher leucine content, and is better digestible than plant-derived protein. Few studies, however, have investigated the influence of protein source on muscle mass in large, population-based studies with wide age ranges. The present study indicates that animal protein, as compared to plant protein, is likely more effective for preserving muscle mass. Meat and dairy are a particularly good source of high quality proteins and furthermore provide bioactive compounds such as cobalamin, vitamin B12 and conjugated linoleic acid. Furthermore, the whey fraction of dairy is particular high in branched chain amino acids [21]. Meat on the other hand provides creatine, carnitine and iron [20]. In line with the high nutrient density of both meat and dairy, Burd et al. [45] demonstrated that both milk and beef consumption increase the post-exercise myofibrillar protein synthetic response, a marker of muscle mass, in young men to similar extent. However, the acute muscle protein synthetic response does not always correlate with long-term changes in muscle mass [46] and these studies did not examine long-term effects of dairy on muscle mass. In the current study creatinine excretion, a long-term marker of muscle mass, was, against expectation, overall only positively associated with meat, but not with dairy intake. Null results in the present study may be explained by a relatively high intake of dairy protein overall in this cohort, limiting the ability to detect a significant association between dairy protein intake and creatinine excretion. In this population, dairy protein intake may have already maximally benefitted muscle mass in older and/or protein-insufficient populations, even in the lowest quartile of intake. Furthermore, in the present study protein from all dairy products was grouped together, whereas dairy products may need to be evaluated per product group to elucidate a potential association with creatinine excretion. Elliot et al. [47] examined the effect of milk ingestion on net muscle protein synthesis following resistance exercise and found that whole milk increased utilization of available amino acids for protein synthesis to a greater extent than fat-free milk that was isocaloric with the whole milk. Whether dairy protein intake has a long-term effect on muscle mass needs to be further clarified. 

Plant protein intake was not positively associated with creatinine excretion in the present study. This is inconsistent with findings from Huang et al. [9]. Kobayashi et al. [48], who examined the association between protein sources (animal or plant) and frailty among elderly Japanese women, found that the association of total protein intake with frailty may be observed regardless of the source of protein.

Recently, questions have been raised regarding the RDA of protein for older adults, who, because of loss of muscle mass, may benefit from protein intakes greater than 0.8 g/kg/day [17,18,49]. The current RDA is based on studies estimating the minimum protein intake necessary to maintain nitrogen balance, not on the maintenance of muscle mass [17]. Protein intakes of 1.0–1.2 g/kg/day have been recommended for the maintenance of muscle mass, whereas intakes of 1.2–1.5 g/kg/day may be necessary in older patients with acute or chronic diseases. Protein intakes up to 2.0 g/kg/day have been recommended for elderly with severe illness or malnutrition [26]. Even though the total protein intake in the present study was higher than the RDA of 0.8 g/kg/day of protein (Table 1, Q1: 0.8–Q4: 1.1), creatinine excretion increased significantly across the quartiles of total protein intake in a dose-dependent way, supporting that increasing daily intakes of total protein may help preserve muscle mass.

Intervention studies [15,25] have clearly demonstrated that combining physical exercise with protein intake has a positive synergistic effect on muscle protein synthesis. In line with an observational study, Morris et al. [22] found that in middle-aged Americans muscle-strengthening exercise is associated with increased muscle mass, if supported by adequate protein intake. Against our expectations, in the present study, a significant interaction between total protein intake and physical activity was observed in women only. Moreover, no linear trend in the associations was found when women were stratified according to physical activity quartiles. The discrepancy between our study and the intervention studies might be explained by the accuracy of the physical activity questionnaire, or by a population-based approach.

Strengths of the present study include the large sample size, which allowed for detailed stratification. Previous similar studies were largely conducted on data from older adults, whereas the present study examined a cohort with a wide range in age. Furthermore, this study examined protein subtypes in relation to 24 h urinary creatinine excretion. Additionally, in this study, various potential confounders were carefully adjusted for. This also study has some limitations, however. Firstly, the cross-sectional design prevents inference of causality between dietary protein intake and creatinine excretion. Secondly, the assessment of dietary intake by means of the FFQ may have resulted in imprecise measurements of nutrient intake, reducing the ability to detect associations between protein intake and creatinine excretion. Thirdly, even though the 24 h urinary creatinine excretion is considered a reliable, useful and inexpensive method of estimating muscle mass, other methods of assessing body composition such as dual energy X-ray absorptiometry may be more accurate [49]. Fourthly, urinary creatinine excretion is influenced to some extent by dietary intake of creatine (derived from meat) and by protein intake [32,36]. However, as we did not adjust for creatine and protein intake, our analyses may overestimate the associations with creatinine excretion, especially for meat intake. Therefore, replication of our results with a muscle mass measurement which is not influenced by dietary intake, such as dual energy X-ray absorptiometry, is needed. Finally, findings in white men and women may not be generalizable to other ethnic groups or races.

Long-term, prospective studies of the influence of different dietary protein sources on muscle mass should be conducted, in order to formulate recommendations for interventions aimed at building and preserving muscle mass in different age groups. 

## 5. Conclusions

To our knowledge, this study is the first cross-sectional study to examine the role of intakes of different dietary protein sources on muscle mass in a large cohort with wide age ranges. The present study suggests that total protein intake, animal protein intake, and in particular fish/meat/egg protein intake are important for building and/or preserving muscle mass in this cohort of adult men and women. Plant protein and dairy protein intake may favor preservation of muscle mass in older women. While a causal relationship between dietary protein intake and muscle mass cannot be established, the results of the present study suggest that low dietary protein intake may be a modifiable risk factor for sarcopenia. The importance of protein intake for muscle mass is confirmed by several intervention studies. However, unlike this study, intervention studies show that the effect of protein intake is dependent on exercise for both men and women and that muscle build up is indifferent of the source of animal protein. Several methodological constraints, such as a relatively high intake of dairy protein compared to protein intake from fish/meat/egg and creatine intake through meat overestimating the association between meat and muscle mass, could explain why in the present study the associations of meat and dairy intake with creatinine excretion, a marker for muscle mass, differed. Intakes of different dietary protein sources should be further studied for their potential to build and preserve muscle mass.

## Figures and Tables

**Table 1:** Baseline characteristics by sex-stratified quartile (Q) of energy-adjusted total protein intake ^1^.

Characteristic	Total (*n* = 76,633)	Q1 (*n* = 19,158)	Q2 (*n* = 19,158)	Q3 (*n* = 19,160)	Q4 (*n* = 19,157)	*p*
**Age** (years)	44.9 ± 12.8	41.5 ± 12.8	44.5 ± 12.7	45.9 ± 12.5	47.7 ± 12.1	<0.001
**Female** (%)	59.2	59.2	59.2	59.2	59.2	0.99
**White** (%)	98.3	97.7	98.3	98.5	98.7	<0.001
**Education** (%)						
-Low	60.8	63.3	59.1	59.4	61.3	<0.001
-Medium	8.9	9.6	9.0	8.6	8.3	<0.001
-High	30.3	27.1	31.9	32.0	30.4	<0.001
**Smoking** (%)						
-Never	45.0	45.5	46.0	45.2	43.1	<0.001
-Former	36.2	30.7	35.8	37.5	40.7	<0.001
-Current	18.9	23.8	18.3	17.2	16.2	<0.001
**Alcohol intake** (%)						
-0 g/day	14.5	16.3	14.4	13.1	14.1	<0.001
->0–<10 g/day	60.0	56.9	59.9	61.7	61.4	<0.001
-≥10–<20g/day	18.4	17.3	18.5	19.0	18.7	<0.001
-≥20 g/day	7.2	9.5	7.2	6.2	5.9	<0.001
**Prevalent health conditions** (%)						
-Diabetes	2.4	1.4	1.9	2.6	3.7	<0.001
-Ischemic heart disease	0.3	0.2	0.3	0.3	0.3	0.008
-Congestive heart failure	0.7	0.5	0.7	0.8	1.0	<0.001
-Hypertension	22.0	17.6	20.8	22.9	26.7	<0.001
-Cerebrovascular accident	0.7	0.7	0.6	0.7	0.9	0.003
-Chronic obstructive pulmonary disease	5.3	5.4	5.0	5.2	5.5	0.059
-Cancer	4.6	3.9	4.3	4.7	5.4	<0.001
**Physical activity**						
-MVPA score	4806 ± 4952	4546 ± 4792	4662 ± 4881	4770 ± 4950	5246 ± 5150	<0.001
**Body composition**						
-Creatinine excretion (mmoL/24 h)	13.3 ± 4.1	13.0 ± 4.2	13.2 ± 4.0	13.4 ± 4.1	13.6 ± 4.3	<0.001
-Waist circumference (cm)	90.3 ± 12.4	88.2 ± 12.3	89.7 ± 12.1	91.0 ± 12.1	92.4 ± 12.5	<0.001
-Height (cm)	174.7 ± 9.4	174.8 ± 9.3	174.6 ± 9.2	174.7 ± 9.4	174.7 ± 9.5	0.271
-Weight (kg)	79.8 ± 15.2	77.3 ± 15.1	79.1 ± 14.8	80.5 ± 15.0	82.2 ± 15.6	<0.001
-BMI (kg/m^2^)	26.1 ± 4.3	25.2± 4.2	25.9 ± 4.2	26.3 ± 4.2	26.9 ± 4.4	<0.001
-BSA (m^2^)	1.95 ± 0.2	1.92 ± 0.2	1.94 ± 0.2	1.95 ± 0.2	1.97 ± 0.2	<0.001
**Blood biomarkers**						
-Hemoglobin (mmoL/L)	8.7 ± 0.8	8.7 ± 0.8	8.7 ± 0.8	8.7 ± 0.8	8.8 ± 0.8	<0.001
-Lymphocyte (%)	34.2 ± 7.7	34.1 ± 7.8	34.1 ± 7.6	34.2 ± 7.7	34.3 ± 7.6	0.028
-Cholesterol (mmoL/L)	5.1 ± 1.0	5.0 ± 1.0	5.1 ± 1.0	5.1 ± 1.0	5.1 ± 1.0	<0.001
-Glucose (mmoL/L)	5.0 ± 0.8	4.9 ± 0.7	5.0 ± 0.8	5.0 ± 0.8	5.1 ± 0.9	<0.001
**Dietary intake**						
-Total energy (kcal/day)	1978 ± 517	2034 ± 624	1911 ± 544	1926 ± 527	2042 ± 573	<0.001
-Fat (% of energy)	34.6	34.2	34.4	34.6	35.0	<0.001
-Carbohydrates (% of energy)	46.8	50.0	47.4	46.0	44.0	<0.001
-Protein (% of energy)	15.4	12.3	14.8	16.1	17.9	<0.001
-Protein (g·kg^−1^·day^−1^)	1.0 ± 0.3	0.8 ± 0.2	0.9 ± 0.2	1.0 ± 0.2	1.1 ± 0.3	<0.001
-Protein (g/day)	74.7 ± 19.8	64.0 ± 17.6	69.4 ± 16.1	75.9 ± 15.7	89.5 ± 19.7	<0.001
-Animal protein (g/day)	44.6 ± 14.1	33.8 ± 10.8	40.0 ± 9.4	46.1 ± 9.1	58.4 ± 13.4	<0.001
-Dairy protein (g/day)	19.5 ± 10.0	13.6 ± 6.8	16.7 ± 7.0	20.0 ± 7.7	27.7 ± 11.5	<0.001
-Fish/meat/egg protein (g/day)	25.1 ± 9.2	20.2 ± 8.4	23.3 ± 7.7	26.1 ± 7.6	30.7 ± 9.6	<0.001
-Plant protein (g/day)	30.2 ± 9.7	30.1 ± 9.9	29.4 ± 9.2	29.9 ± 9.3	31.2 ± 10.3	<0.001
-Alcohol (g/day)	7.0 ± 8.7	7.6 ± 10.3	7.0 ± 8.4	6.9 ± 7.9	6.7 ± 7.8	<0.001

^1^ Results are mean ± standard deviation. Q = quartile.

**Table 2:** Adjusted least square means for creatinine excretion (mmoL/24 h) among the different quartiles of energy-adjusted protein intake ^1^.

	Quartiles of Protein Intake				
**Model 1** ^2^					
	**Q1**	**Q2**	**Q3**	**Q4**	***p*-trend**
**Men**					
Total protein	16.38 (16.31–16.45)	16.60 (16.52–16.67) *	16.75 (16.67–16.82) *	16.98 (16.91–17.05) *	<0.001
Animal protein	16.37 (16.29–16.44)	16.59 (16.51–16.66) *	16.77 (16.70–16.84) *	16.98 (16.91–17.06) *	<0.001
-Dairy protein	16.72 (16.65–16.79)	16.66 (16.59–16.73)	16.64 (16.57–16.71)	16.68 (16.61–16.76)	0.442
-Fish/meat/egg protein	16.19 (16.11–16.26)	16.54 (16.47–16.61) *	16.78 (16.71–16.86) *	17.20 (17.13–17.27) *	<0.001
Plant protein	16.68 (16.61–16.75)	16.69 (16.62–16.76)	16.65 (16.58–16.72)	16.68 (16.61–16.75)	0.893
**Women**					
Total protein	10.77 (10.73–10.81)	11.01 (10.97–11.05) *	11.10 (11.06–11.14) *	11.17 (11.13–11.21) *	<0.001
Animal protein	10.78 (10.74–10.82)	11.00 (10.95–11.03) *	11.10 (11.06–11.14) *	11.19 (11.14–11.23) *	<0.001
-Dairy protein	11.00 (10.96–11.05)	11.00 (10.95–11.03)	11.03 (10.99–11.07)	11.04 (10.99–11.08)	0.392
-Fish/meat/egg protein	10.65 (10.61–10.69)	11.01 (10.97–11.05) *	11.12 (11.08–11.16) *	11.27 (11.23–11.31) *	<0.001
Plant protein	11.01 (10.96–11.05)	11.03 (10.99–11.07)	10.99 (10.95–11.03)	11.03 (10.99–11.07)	0.474
**Model 2** ^3^					
	**Q1**	**Q2**	**Q3**	**Q4**	***p*-trend**
**Men**					
Total protein	16.36 (16.29–16.43)	16.62 (16.55–16.69) *	16.77 (16.70–16.84) *	16.95 (16.88–17.02) *	<0.001
Animal protein	16.34 (16.27–16.41)	16.61 (16.54–16.68) *	16.79 (16.72-16.87) *	16.96 (16.89-17.03) *	<0.001
-Dairy protein	16.70 (16.62–16.77)	16.70 (16.63–16.77)	16.67 (16.60-16.74)	16.64 (16.57-16.71)	0.653
-Fish/meat/egg protein	16.15 (16.08–16.22)	16.55 (16.47–16.62) *	16.81 (16.74-16.88) *	17.20 (17.13-17.28) *	<0.001
Plant protein	16.66 (16.58–16.73)	16.72 (16.650–16.79)	16.68 (16.61-16.75)	16.64 (16.57-16.72)	0.464
**Women**					
Total protein	10.77 (10.73–10.81)	11.03 (10.99–11.07) *	11.11 (11.07–11.15) *	11.14 (11.10–11.19) *	<0.001
Animal protein	10.77 (10.73–10.81)	11.01 (10.97–11.05) *	11.11 (11.07–11.15) *	11.17 (11.12–11.21) *	<0.001
-Dairy protein	10.98 (10.94–11.02)	11.02 (10.98–11.06)	11.05 (11.01–11.09)	11.01 (10.97–11.05)	0.112
-Fish/meat/egg protein	10.66 (10.62–10.70)	11.00 (10.96–11.04) *	11.12 (11.08–11.16) *	11.27 (11.23–11.31) *	<0.001
Plant protein	10.98 (10.94–11.02)	11.06 (11.02–11.10) *	11.02 (10.98–11.06)	11.00 (10.96–11.04)	0.032

^1^ Results are least square means for creatinine excretion (mmoL/24 h) ± 95% Confidence Interval (CI). Q = quartile. ^2^ Model 1 was adjusted for age and body surface area. Animal protein and plant protein intake were adjusted for each other in the same model. ^3^ Model 2 was adjusted for variables in model 1 plus total energy intake, smoking status, alcohol consumption (categorical), physical activity, prevalent health conditions (i.e., diabetes, ischemic heart disease, congestive heart failure, cerebrovascular accident, chronic obstructive pulmonary disease, cancer, and hypertension), hemoglobin, and lymphocyte percentage. Animal protein and plant protein intake were adjusted for each other in the same model. * Significantly different from Q1, *p* < 0.05 (ANCOVA with Bonferroni test).

**Table 3:** Adjusted regression coefficients (and 95% CI) for creatinine excretion (mmoL/24 h) per unit of energy-adjusted protein intake ^1^.

	Men		Women	
	β (95% CI)	*p*	β (95% CI)	*p*
**Model 1** ^2^				
Total protein	0.022 (0.018–0.025)	<0.001	0.015 (0.013–0.017)	<0.001
Animal protein	0.023 (0.019–0.026)	<0.001	0.015 (0.013–0.017)	<0.001
Dairy protein	0.001 (–0.003–0.005)	0.633	0.001 (–0.002–0.003)	0.637
Fish/meat/egg protein	0.051 (0.047–0.055)	<0.001	0.033 (0.030–0.036)	<0.001
Plant protein	0.002 (–0.004–0.009)	0.455	0.004 (–0.001–0.009)	0.091
**Model 2** ^3^				
Total protein	0.021 (0.018–0.025)	<0.001	0.014 (0.012–0.017)	<0.001
Animal protein	0.022 (0.019–0.026)	<0.001	0.015 (0.012–0.017)	<0.001
Dairy protein	–0.001 (–0.005–0.003)	0.685	0.000 (–0.003–0.002	0.882
Fish/meat/egg protein	0.052 (0.048–0.057)	<0.001	0.033 (0.030–0.036)	<0.001
Plant protein	0.001 (–0.005–0.008)	0.657	0.004 (–0.001–0.009)	0.158

^1^ Results are β (95% Confidence Interval (CI)). ^2^ Model 1 was adjusted for age and body surface area. Animal protein and plant protein intake were adjusted for each other in the same model. ^3^ Model 2 was adjusted for variables in model 1 plus total energy intake, smoking status, alcohol consumption (categorical), physical activity, prevalent health conditions (i.e., diabetes, ischemic heart disease, congestive heart failure, cerebrovascular accident, chronic obstructive pulmonary disease, cancer, and hypertension), hemoglobin, and lymphocyte percentage. Animal protein and plant protein intake were adjusted for each other in the same model.

**Table 4:** Adjusted regression coefficients (and 95% CI) for creatinine excretion (mmoL/24 h) per unit of energy-adjusted protein intake among the different age groups ^1^.

	Age Group						
	18–25	26–35	36–45	46–55	56–65	66–75	>75
**Model 1** ^2^							
**Men, *n***	1591	5453	8864	8610	4464	1998	298
Total protein	0.020 (0.006–0.034) **	0.026 (0.019–0.034) *	0.024 (0.018–0.030)*	0.017 (0.010–0.023) *	0.021 (0.013–0.028) *	0.013 (0.002–0.024) **	0.003 (−0.026–0.033)
Animal protein	0.022 (0.008–0.037) **	0.027 (0.003–0.032) *	0.026 (0.020–0.032) *	0.018 (0.011–0.024) *	0.021 (0.013–0.029) *	0.013 (0.002–0.024) **	0.004 (−0.025–0.034)
-Dairy protein	−0.007 (−0.025–0.011)	0.000 (−0.010–0.010)	0.002 (−0.006–0.009)	−0.001 (−0.009–0.006)	0.008 (−0.001–0.017) ***	−0.007 (−0.020–0.006)	−0.032 (−0.066–0.003) ***
-Fish/meat/egg protein	0.054 (0.035–0.073) *	0.055 (0.046–0.065) *	0.057 (0.049–0.066)*	0.047 (0.038–0.056) *	0.039 (0.028–0.049) *	0.041 (0.026–0.055) *	0.054 (0.014–0.093) **
Plant protein	0.003 (−0.024–0.031)	0.018 (0.003–0.032) **	−0.004 (−0.016–0.009)	−0.006 (−0.020–0.007)	−0.008 (−0.024–0.008)	−0.025 (−0.049–0.001) **	−0.015 (−0.078–0.047)
**Women, *n***	3390	7570	13156	12509	6151	2259	320
Total protein	0.015 (0.006–0.024) *	0.018 (0.013–0.024) *	0.010 (0.006–0.014) *	0.015 (0.011–0.019) *	0.015 (0.010–0.020) *	0.022 (0.014–0.030) *	0.029 (0.010–0.048) **
Animal protein	0.016 (0.007–0.025) *	0.019 (0.013–0.025) *	0.010 (0.006–0.015) *	0.015 (0.011–0.019) *	0.014 (0.009–0.019) *	0.022 (0.013–0.030) *	0.028 (0.009–0.047) **
-Dairy protein	−0.002 (–0.013–0.010)	0.004 (−0.003–0.012)	−0.004 (−0.009–0.001)	0.000 (−0.004–0.005)	0.003 (−0.003–0.009)	0.013 (0.002–0.023) **	0.017 (−0.008–0.041)
-Fish/meat/egg protein	0.034 (0.022–0.045) *	0.034 (0.026–0.041) *	0.029 (0.023–0.034) *	0.036 (0.030–0.041) *	0.029 (0.023–0.036) *	0.034 (0.023–0.045) *	0.039 (0.014–0.064) **
Plant protein	0.005 (−0.015–0.024)	0.005 (−0.007–0.018)	−0.001 (−0.011–0.008)	0.008 (−0.001–0.018) ***	−0.007 (−0.020–0.005)	0.018 (−0.003–0.040) ***	0.050 (0.003–0.098) **
**Model 2** ^3^							
**Men, *n***	1591	5453	8864	8610	4464	1998	298
Total protein	0.020 (0.006–0.034) **	0.027 (0.020–0.035) *	0.024 (0.018–0.031) *	0.016 (0.010–0.023) *	0.019 (0.011–0.027) *	0.013 (0.001–0.024) **	0.004 (−0.027–0.035)
Animal protein	0.022 (0.008–0.036) **	0.028 (0.020–0.035) *	0.026 (0.020–0.033) *	0.017 (0.011–0.024) *	0.019 (0.011–0.027) *	0.012 (0.001–0.024) **	0.005 (−0.026–0.036)
-Dairy protein	−0.006 (−0.025–0.012)	0.001 (−0.009–0.011)	0.001 (−0.007–0.008)	−0.004 (−0.011–0.004)	0.003 (−0.007–0.012)	−0.012 (−0.025–0.002) ***	−0.033 (−0.069–0.003) ***
-Fish/meat/egg protein	0.052 (0.033–0.071) *	0.056 (0.046–0.065) *	0.059 (0.051–0.068) *	0.048 (0.039–0.057) *	0.041 (0.030–0.052) *	0.042 (0.027–0.057) *	0.055 (0.015–0.095) **
Plant protein	0.004 (−0.024–0.033)	0.020 (0.005–0.034) **	−0.004 (−0.017–0.008)	−0.009 (−0.022–0.005)	−0.015 (−0.031–0.002) ***	−0.025 (−0.050–0.001) ***	−0.013 (−0.080–0.053)
**Women, *n***	3390	7570	13156	12509	6151	2259	320
Total protein	0.017 (0.008–0.026) *	0.019 (0.013–0.025)*	0.011 (0.007–0.015) *	0.014 (0.010–0.018) *	0.013 (0.008–0.017) *	0.019 (0.010–0.027) *	0.022 (0.002–0.041) **
Animal protein	0.017 (0.008–0.026) *	0.020 (0.014–0.026) *	0.011 (0.007–0.015) *	0.014 (0.010–0.018) *	0.011 (0.006–0.016) *	0.018 (0.010–0.027) *	0.021 (0.002–0.041) **
-Dairy protein	0.001 (−0.010–0.013)	0.005 (−0.002–0.013)	−0.004 (−0.009–0.001)	−0.002 (−0.006–0.003)	−0.002 (−0.008–0.004)	0.007 (−0.003–0.018)	0.004 (−0.021–0.030)
-Fish/meat/egg protein	0.033 (0.022–0.045) *	0.035 (0.027–0.042) *	0.030 (0.024–0.035) *	0.035 (0.030–0.041) *	0.029 (0.022–0.035) *	0.032 (0.021–0.044) *	0.038 (0.013–0.064) **
Plant protein	0.009 (−0.011–0.029)	0.007 (−0.006–0.019)	−0.001 (−0.011–0.008)	0.007 (−0.003–0.016)	−0.016 (−0.028–0.003) **	0.015 (−0.007–0.037)	0.030 (−0.021–0.081)

^1^ Results are β (95% Confidence Interval (CI)). *n* = number. ^2^ Model 1 was adjusted for age and body surface area. Animal protein and plant protein intake were adjusted for each other in the same model. ^3^ Model 2 was adjusted for variables in model 1 plus total energy intake, smoking status, alcohol consumption (categorical), physical activity, prevalent health conditions (i.e., diabetes, ischemic heart disease, congestive heart failure, cerebrovascular accident, chronic obstructive pulmonary disease, cancer, and hypertension), hemoglobin, and lymphocyte percentage. Animal protein and plant protein intake were adjusted for each other in the same model. * *p* < 0.001, ** *p* < 0.05, *** *p* < 0.10.

## Abbreviations

ANCOVA	Analysis of covariance
ANOVA	Analysis of variance
BMI	Body mass index
BSA	Body surface area
CI	Confidence interval
COPD	Chronic obstructive pulmonary disease
DIAAS	Digestible Indispensable Amino Acid Score
FAO	Food and Agriculture Organization
FFQ	Food frequency questionnaire
MET	Metabolic equivalent
MVPA	Moderate-intensity and vigorous-intensity physical activity
Q	Quartile
RDA	Recommended dietary allowance
SD	Standard deviation
SQUASH	Short questionnaire to assess health-enhancing physical activity
UMCG	University Medical Center Groningen
